# Effects of starting powder and thermal treatment on the aerosol deposited BaTiO_3_ thin films toward less leakage currents

**DOI:** 10.1186/1556-276X-9-435

**Published:** 2014-08-27

**Authors:** Zhao Yao, Cong Wang, Yang Li, Hong-Ki Kim, Nam-Young Kim

**Affiliations:** 1 Department of Electronic Engineering, Kwangwoon University, 20 Gwangun-Ro, Nowon-gu, Seoul 139-701, Republic of Korea; 2 Department of Electronic Materials Engineering, Kwangwoon University, 20 Gwangun-Ro, Nowon-gu, Seoul 139-701, Republic of Korea

**Keywords:** Sulfur hexafluoride, Post-annealing treatment, Inductively coupled plasma etching, Barium titanate

## Abstract

To prepare high-density integrated capacitors with low leakage currents, 0.2-μm-thick BaTiO_3_ thin films were successfully deposited on integrated semiconductor substrates at room temperature by the aerosol deposition (AD) method. In this study, the effects of starting powder size were considered in an effort to remove macroscopic defects. A surface morphology of 25.3 nm and an interface roughness of less than 50 nm were obtained using BT-03B starting powder. The nano-crystalline thin films achieved after deposition were annealed at various temperatures to promote crystallization and densification. Moreover, the influence of rapid thermal annealing process on the surface morphology and crystal growth was evaluated. As the annealing temperature increased from room temperature to 650°C, the root mean square (RMS) roughness decreased from 25.3 to 14.3 nm. However, the surface was transformed into rough performance at 750°C, which agreed well with the surface microstructure trend. Moreover, the crystal growth also reveals the changes in surface morphology via surface energy analysis.

## Background

Recently, to meet the modern communication system demands of miniaturization and high frequency, high-density integrated capacitors have attracted increasing industry interest, which has been driven by thin-film integrated passive devices (IPDs) [1-3], electromagnetic interference (EMI) protection [4], high-electron-mobility transistor (HEMT) input-/output-matching circuit blocks [5], and digital and mixed signal applications [6]. Several semiconductor technologies, such as low-temperature co-firing ceramics (LTCC) [7] and sputtering [8], can be used to fabricate materials with high relative permittivity. However, both LTCC and sputtering need sintering at approximately 850°C to form the desired crystallite structure, which is a critical problem for embedding passive devices. Consequently, a new, green, and environmentally friendly approach called aerosol deposition (AD), which can deposit ceramic films at room temperature, has attracted great interest. The pioneering work was published in 2001 [9], and various ceramic films fabricated by AD have been studied quite intensively in recent years.

In previous research, ferroelectric BaTiO_3_ was employed in high-density embedded decoupling capacitors using the AD method. BaTiO_3_ films with thicknesses of 0.1 to 2.2 μm were deposited on Cu and stainless steel (SUS) substrates [10-13]. The BaTiO_3_ films with a thickness of less than 0.5 μm on Cu substrates and 0.2 μm on SUS substrates exhibited conductor properties because of their high leakage currents. The leakage current mechanisms for aerosol-deposited BaTiO_3_ thin films and the causes of the high leakage currents were determined in previous research [10,12]. However, the densification mechanism of BaTiO_3_ films deposited by AD has yet to be identified.

In this study, we applied 0.2-μm-thick BaTiO_3_ thin films deposited by AD onto an integrated substrate suitable for thin-film IPDs. To overcome the macroscopic defects and rough interface between the BaTiO_3_ films and substrates, the influence of starting powders with difference particle sizes was investigated by scanning electron microscopy (SEM) and focused ion beam (FIB). In addition, the densification of AD-deposited BaTiO_3_ thin films and stronger particle-to-particle bonding could be obtained using rapid thermal annealing treatment. The surface morphology of post-annealed BaTiO_3_ thin films was examined using atom force microscopy (AFM) to reveal the effect of rapid thermal annealing (RTA) treatment on leakage currents.

## Methods

The AD method is a very attractive deposition process for integrating ceramic thin films. During the deposition process, the raw particles are mixed with a N_2_ carrier gas to form an aerosol flow and then ejected through a nozzle and coated onto the substrate in the deposition chamber at room temperature. The detailed fabrication apparatus has been described in elsewhere [14]. The BaTiO_3_ thin films were successfully deposited on Pt/Ti/SiO_2_/Si integrated substrates with a thickness of 200 nm and a deposition area of 10 × 10 mm^2^ using a similar AD apparatus in this paper. The thickness of the Pt/Ti layer is 150/10 nm. During the deposition process, to clarify the influence of the starting powder on the morphology of the bottom Pt interface, different BaTiO_3_ powders BT-045J and BT-03B (Samsung Fine Chemicals Co., Ltd., Ulsan, South Korea) with particle sizes of 0.45 and 0.30 μm, respectively, were used as starting powders. The surfaces of the as-deposited thin films were evaluated using SEM (S-4300SE; Hitachi Ltd, Tokyo, Japan), and the cross-section of the interface between the BaTiO_3_ thin films and Pt substrate deposited using different starting powders was observed using a FIB system (Nova 600 Nanolab, FEI, Hillsboro, OR, USA).

**Figure 1:**
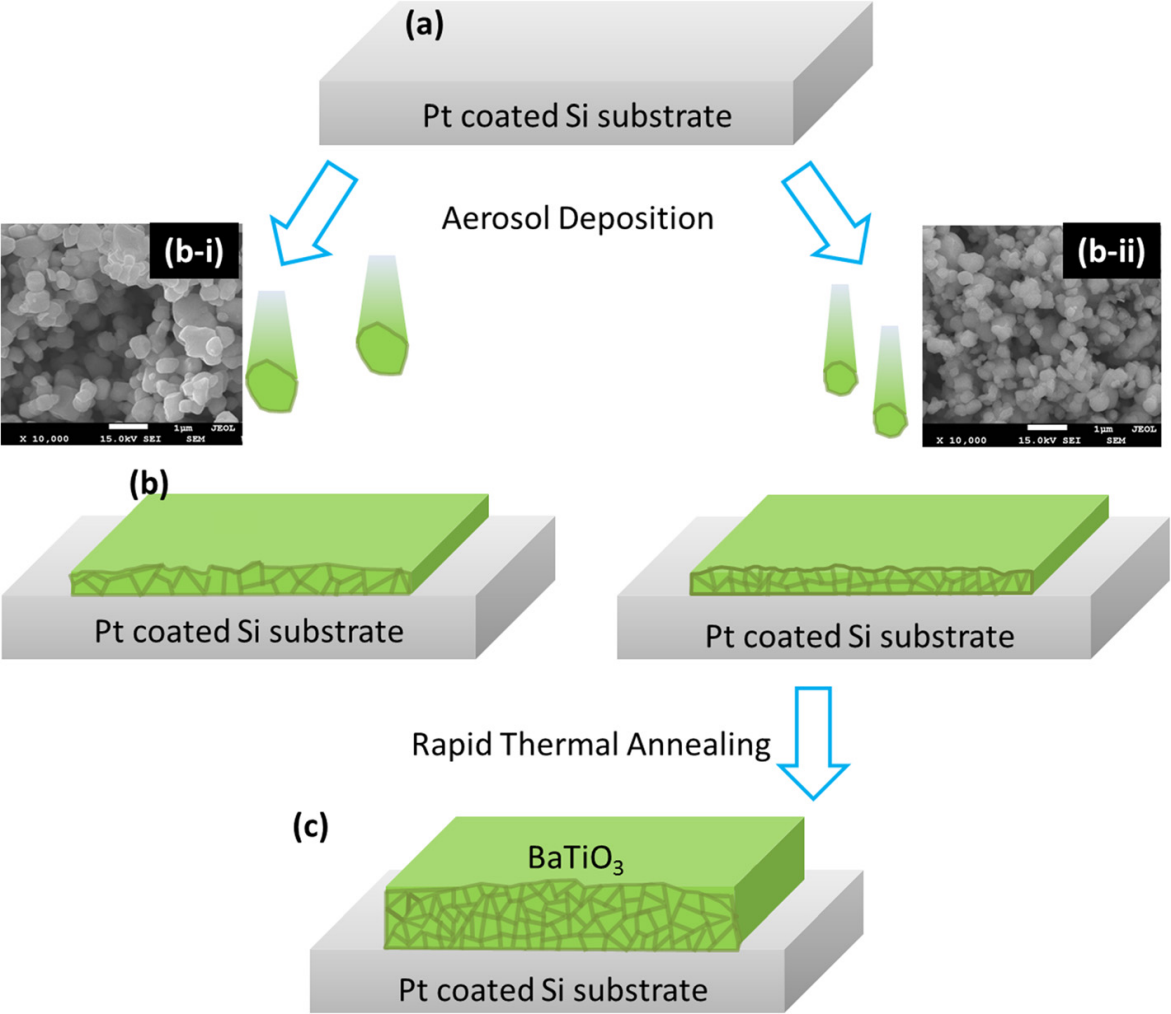
**Fabrication process. (a)** Silicon substrate coated with Pt/Ti (150/10 nm) is cleaned with acetone and deionized water; **(b)** schematic of growth of BaTiO_3_ thin films by aerosol deposition using different starting powder; inset pictures show the SEM images of the starting powder **(b-i)** BT-045J and **(b-ii)** BT-03B (with a particle size of 0.45 and 0.3 μm, respectively); and **(c)** 0.2-μm-thick as-deposited BaTiO_3_ thin films annealed at 550, 650, and 750°C for 60 s.

To enhance the crystal growth, the as-deposited thin films were annealed for 60 s in N_2_ ambient by RTA (KVR-3006 T, Korea Vacuum Tech., Gyeonggi, South Korea). The annealing temperature was varied between 550°C and 750°C in 100°C steps. Figure 1 schematically shows the fabrication process. After RTA treatment, the post-annealed thin films were analyzed by X-ray diffraction (XRD; ATX-G, Rigaku, Tokyo, Japan) using Cu Kα radiation (*λ* = 0.154 nm) with a power of 18 kW. Moreover, the surface morphology of the post-annealed samples was measured by AFM (XE-100, PSIA Co., Sungnam, South Korea).

## Results and discussion

### Surface roughness

In our previous work, BaTiO_3_ films of 0.1 to 2.2 μm in thickness were deposited on Cu and SUS substrate by the AD method. All of the samples with thicknesses of less than 0.5 μm on Cu substrates and 0.2 μm on SUS substrates were electrically shorted, which can be a result of high leakage currents caused by macroscopic defects and rough interfaces between films and substrates [10].

**Figure 2:**
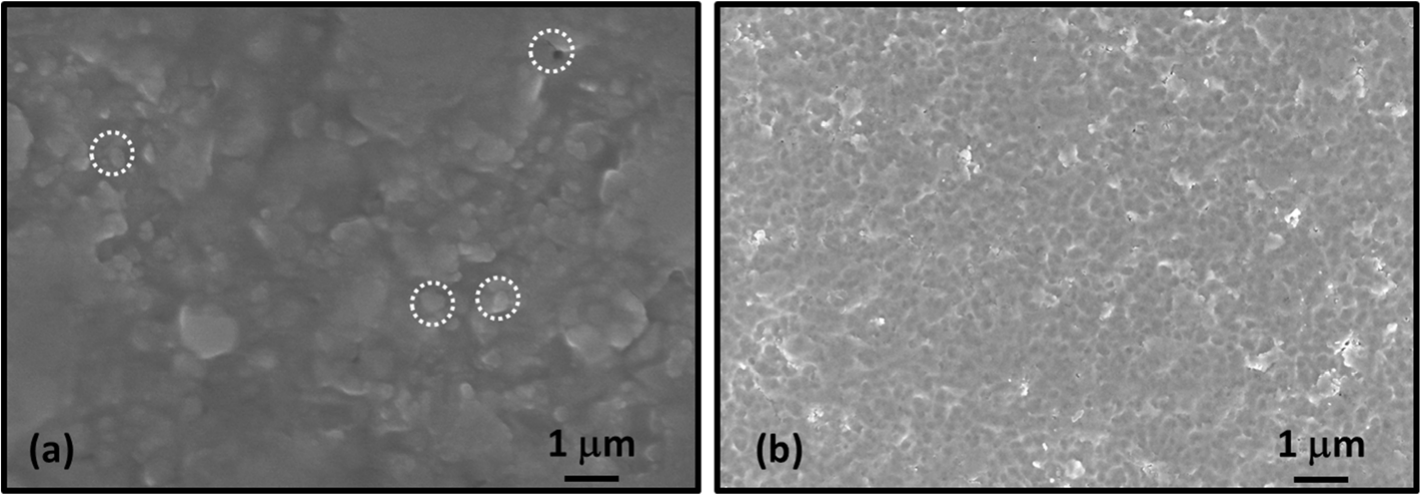
**SEM images of the surface morphology of BaTiO**_**3 **_**thin films deposited. (a)** BT-045 J starting powder and **(b)** BT-03B starting powder**.**

In this study, 0.2-μm-thick BaTiO_3_ films were fabricated on platinum-coated silicon substrates to apply the AD-deposited BaTiO_3_ thin films in integrated high-K metal-isolator-metal capacitors. Figure 2a,b shows the SEM images of the surface morphologies of BaTiO_3_ thin films fabricated on platinum-coated substrate using BT-045J and BT-03B starting powders, respectively. As shown in Figure 2a, macroscopic defects such as craters and incompletely crushed particles were observed, which were considered to be one of the main causes of the high leakage currents. In contrast, BaTiO_3_ thin films deposited using BT-03B starting powder exhibited a dense surface structure with fewer craters and large particles. It was confirmed that the small starting powder could produce a smoother surface with fewer craters and incompletely crushed particles, thereby decreasing the leakage current [12].

### Interface between BaTiO_3_ thin films and substrates

**Figure 3:**
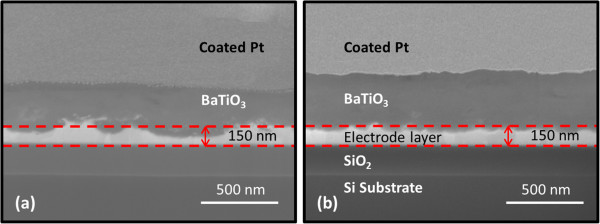
**FIB cross-section images of 0.2-μm-thick BaTiO**_**3 **_**thin films on platinum-coated substrates fabricated. (a)** BT-045J with a particle size of 0.45 μm and **(b)** BT-03B with a particle size of 0.30 μm.

Previous studies, such as [10] and [12], only address the interface between films and Cu or SUS substrate with a minimum interface roughness of 50 to 100 nm. When BaTiO_3_ thin films thickness decreases to less than 200 nm, it would cause a high field concentration, bringing about high leakage currents. In this study, the effect of starting powder size on the interface roughness was demonstrated by FIB. The interface between the films deposited by BT-045J starting powder and the substrates, as shown in Figure 3a, was very rough compared with that using BT-03B starting powder, as shown in Figure 3b. The interface roughness of the films deposited using BT-045J was approximately 70 nm, compared with a roughness of less than 50 nm for the films deposited using BT-03B. These results indicate that larger particles with greater kinetic energy roughen the platinum thin films on the silicon substrates much more severely during impact with the substrates. Thus, interface between the films deposited by BT-045J was rougher than that obtained using BT-03B starting powder.

### Effect of rapid thermal annealing on surface morphology and crystal growth

**Figure 4:**
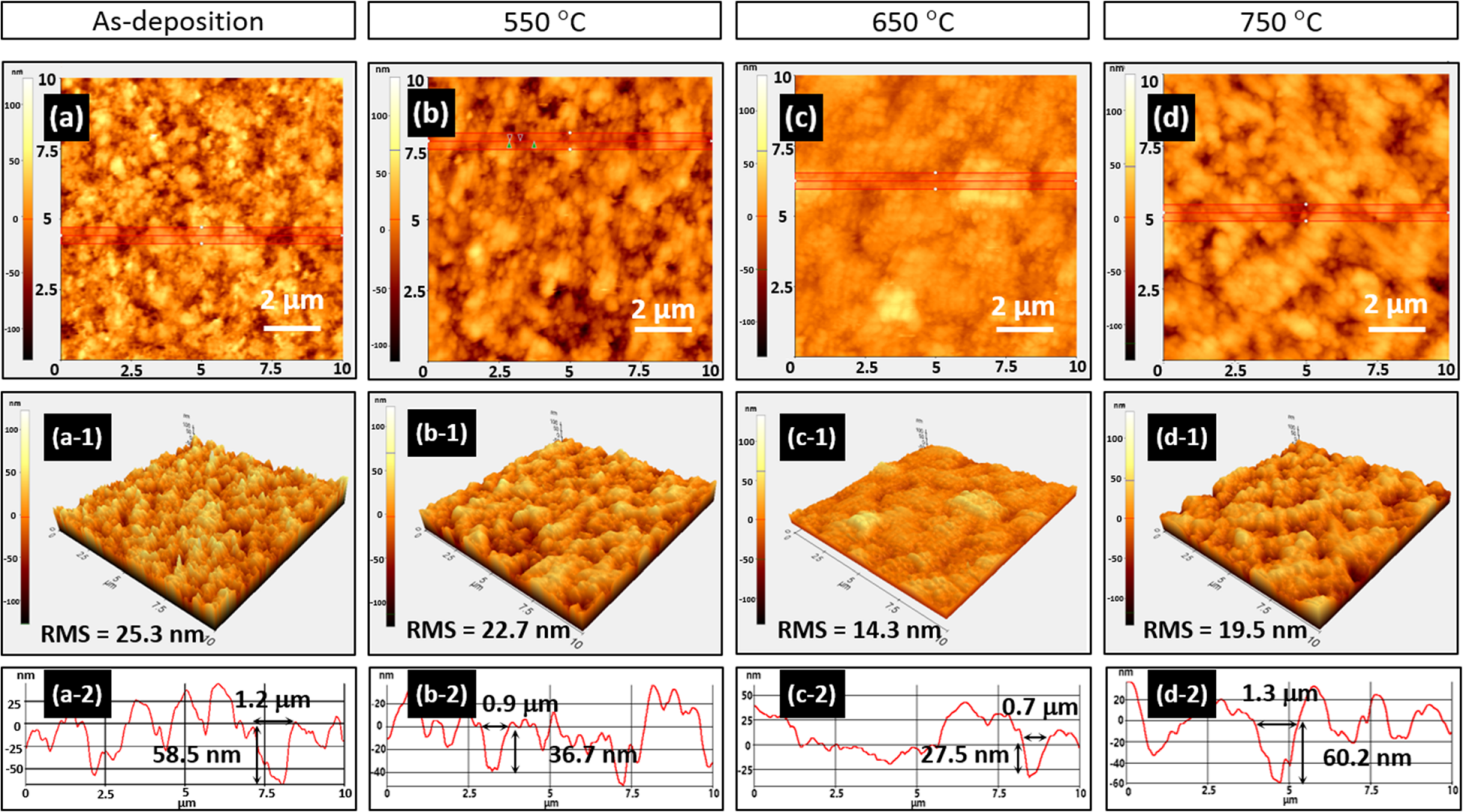
**AFM surface morphology of the as-deposited BaTiO**_**3 **_**thin film. (a)** 2D view, **(a-1)** 3D view, and **(a-2)** line profile of the selected area in the AFM images with a scan area of 10 × 10 μm^2^. AFM images of BaTiO_3_ thin films annealed for 60 s at different temperatures: 550°C **(b)**, 650°C **(c)**, and 750°C **(d)**. The corresponding 3D view and line profiles of the selected areas are shown in **(b-1)** and **(b-2)** and **(c-1)** and **(c-2)** as well as **(d-1)** and **(d-2)**, respectively.

Based on the above-mentioned statement, the macroscopic defects and rough interface effect could be ameliorated by means of BT-03B starting powder to reduce the leakage current. However, it was difficult to form dense films using small particles with weak particle-to-particle bonding as the starting powder [15]. Therefore, we apply RTA treatment in this study and investigate the effects of RTA processing on the surface morphology of AD-deposited BaTiO_3_ thin films. Figure 4 shows 10 × 10 μm^2^ AFM images of 2-D views, 3-D views, and selected area surface profiles of the as-deposited films fabricated by BT-03B starting powder (a) and the post-annealed films processed at different temperatures: 550°C (b), 650°C (c), and 750°C (d). Comparing Figure 4a,b,c, which presents 3-D views of the film surface morphology, it can be noted that the surface becomes smoother and the RMS value decreases as the RTA temperature increases from room temperature to 650°C. In contrast, Figure 4d reveals that the RMS value increased and agglomerates were present on the surface. Moreover, the line profiles of the selected area are shown in Figure 4 (a-2) to (d-2), which indicated the change in both the diameter and depth of the craters on the surface, which follow the trend in Figure 4a,b,c,d. Figure 4 (a-2) shows the craters on the as-deposited films, which have a diameter of 1.2 μm and a depth of 58.5 nm, and the smaller craters observed after RTA treatment at 650°C, which have a diameter of 0.7 μm and a depth of 27.5 nm. However, as shown in Figure 4 (d-2), at 750°C, larger craters with a diameter of 1.3 μm and a depth of 60.2 nm appeared on the surface of the thin film. It was implied that the low surface roughness achieved at 650°C may be due to the microstructure on the surface.

**Figure 5:**
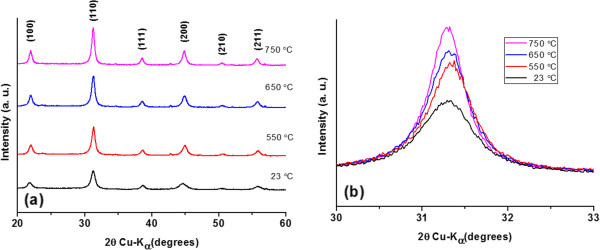
**XRD profile of the AD-deposited BaTiO**_**3 **_**thin films deposited on platinum-coated substrates. (a)** Annealing at various temperatures and **(b)** 110 peak between 30° and 33°.

To explain this finding, the crystal structure was analyzed by XRD to confirm the crystal growth after RTA treatment. As the temperature increased from room temperature to 750°C, all of the XRD profiles, as shown in Figure 5a, confirmed that both the as-deposited and post-annealed BaTiO_3_ thin films have a cubic phase with a single perovskite structure [16]. Figure 5b shows an enlargement of the 110 main peak of the as-deposited BaTiO_3_ thin films and post-annealed thin films at various temperatures. It can be noted that the spectral peaks do not shift in position but do broaden. Moreover, the crystallite size of AD-deposited BaTiO_3_ thin films on platinum-coated substrates at room temperature calculated by Scherrer's equation was 11.3 nm. After post-annealing at 550, 650, and 750°C, the crystallite sizes were 14.5, 16.3, and 17.5 nm, respectively. Similar phenomenon was reported by Kim et al. [17] for BaTiO_3_ films sintered at 800, 900, and 1,000°C. Combined with the surface morphology after RTA, this finding can be explained by surface energy theory as follows [18]. After the RTA treatment, the surface energy would be reduced by combining individual particles into a bulk with a solid interface to enhance the particle-to-particle bonding. As the RTA temperature increased from room temperature to 650°C, volume diffusion dominates the annealing process, resulting in densification and removal of the pores in bulk films. Therefore, a smoother surface morphology and reduction in crater diameter were observed during this process. However, when the annealing temperature was 750°C, cross grain boundary diffusion became significant, leading to a change in surface roughness and microstructure.

## Conclusions

In this study, BaTiO_3_ thin films with thickness of 0.2 μm were deposited on platinum-coated silicon substrates at room temperature by AD. Different thin films deposited using starting powders of various sizes were investigated, and the results confirmed that the macroscopic defects such as pores and incompletely crushed particles could be reduced by employing BT-03B starting powder. An interface roughness of less than 50 nm and a minimum surface roughness of 14.3 nm were obtained after RTA treatment at 650°C. As the annealing temperature increased from room temperature to 650°C, the calculated crystalline size increased from 11.3 to 16.3 nm. Thus, the surface morphology and the densification of AD-deposited BaTiO_3_ thin films can be controlled by appropriate choice of RTA temperature to achieve a low leakage current.

## Competing interests

The authors declare that they have no competing interests.

## Authors’ contributions

ZY participated in the conception of this study, managed the whole study, and drafted the manuscript. H-KK, YL, and CW carried out the fabrication and measurement. As the corresponding author, N-YK managed the main conception, guided the research, and revised the manuscript. All authors read and approved the final manuscript.
